# Transcutaneous electrical nerve stimulation attenuates CFA-induced hyperalgesia and inhibits spinal ERK1/2-COX-2 pathway activation in rats

**DOI:** 10.1186/1472-6882-13-134

**Published:** 2013-06-15

**Authors:** Jun-Fan Fang, Yi Liang, Jun-Ying Du, Jian-Qiao Fang

**Affiliations:** 1 Department of Neurobiology & Acupuncture Research, the Third Clinical College, Zhejiang Chinese Medical University, Hangzhou, China

**Keywords:** Inflammatory pain, Transcutaneous electrical nerve stimulation, Anti-inflammatory pain, CFA, ERK1/2, COX-2, PGE_2_, Signal transduction pathway

## Abstract

**Background:**

Transcutaneous electrical nerve stimulation (TENS) is a non-pharmacologic treatment for pain relief. In previous animal studies, TENS effectively alleviated Complete Freund’s Adjuvant (CFA)- or carrageenan-induced inflammatory pain. Although TENS is known to produce analgesia via opioid activation in the brain and at the spinal level, few reports have investigated the signal transduction pathways mediated by TENS. Prior studies have verified the importance of the activation of extracellular signal-regulated kinase (ERK) signal transduction pathway in the spinal cord dorsal horn (SCDH) in acute and persistent inflammatory pains. Here, by using CFA rat model, we tested the efficacy of TENS on inhibiting the expressions of p-ERK1/2 and of its downstream cyclooxygenase-2 (COX-2) and the level of prostaglandin E_2_ (PGE_2_) at spinal level.

**Methods:**

Rats were randomly divided into control, model and TENS groups, and injected subcutaneously with 100 μl CFA or saline in the plantar surface of right hind paw. Rats in the TENS group were treated with TENS (constant aquare wave, 2 Hz and 100 Hz alternating frequencies, intensities ranging from 1 to 2 mA, lasting for 30 min each time) at 5 h and 24 h after injection. Paw withdrawal thresholds (PWTs) were measured with dynamic plantar aesthesiometer at 3d before modeling and 5 h, 6 h, and 25 h after CFA injection. The ipsilateral sides of the lumbar spinal cord dosral horns were harvested for detecting the expressions of p-ERK1/2 and COX-2 by western blot analysis and qPCR, and PGE_2_ by ELISA.

**Results:**

CFA-induced periphery inflammation decreased PWTs and increased paw volume of rats. TENS treatment significantly alleviated mechanical hyperalgesia caused by CFA. However, no anti-inflammatory effect of TENS was observed. Expression of p-ERK1/2 protein and COX-2 mRNA was significantly up-regualted at 5 h and 6 h after CFA injection, while COX-2 and PGE_2_ protein level only increased at 6 h after modeling. Furthermore, the high expression of p-ERK1/2 and COX-2, and over-production of PGE_2_ induced by CFA, were suppressed by TENS administration.

**Conclusions:**

TENS may be an effective therapy in controlling inflammatory pain induced by CFA. Its analgesic effect may be associated with the inhibition of activation of the spinal ERK1/2-COX-2 pathway.

## Background

Inflammatory pain decreases the quality of life of patients and is thus a major health care problem. Inflammation-induced pain is a complex pathological process occurring in both central nervous system (CNS) and peripheral nervous system. Recent studies have revealed that, the mitogen activated protein kinases (MAPKs) family, located in the spinal cord, plays pivotal roles in regulating inflammatory pain [1].

Extracellular signal-regulated kinase (ERK), the first member identified from the MAPK family, was initially known as a primary effecter of growth factor receptor signaling. However, increasing evidences have also pinpointed ERK as an important mediator in adult neuronal plasticity [2]. Ji et al. (1999) have shown that phosphorylation (activation) of ERK in the spinal cord dorsal horn (SCDH) is depended upon nociceptive activity [3]. Studies addressing the role of ERK1/2 in inflammatory pain have demonstrated that ERK1/2 activation is induced in SCDH by: hind paw inflammation with formalin [4], Complete Freund’s Adjuvant (CFA) [5,6], scorpion BmK venom [7], by chronic bladder inflammation [8], and by monoarthritis in the ankle [9], all contributes to inflammation-induced hyperalgesia and allodynia. Further studies have also revealed that intrathecal injection of specific MEK (ERK1/2’s upstream MAPK kinase) inhibitor, significantly reduces the heat and mechanical hypersensitivity induced by peripheral inflammation [4-9]. Overall, these findings indicate that ERK1/2 activation, followed by COX-2 [10], plays an important role in the generation of inflammatory pain, and thus would be a suitable therapeutic target for inflammatory pain treatment. This hypothesis is strengthened by the fact that COX-2 was believed to contribute to inflammatory pain for a long time.

Transcutaneous electrical nerve stimulation (TENS) is an effective pain treatment method significantly attenuating multiple types of pain, such as inflammatory and neuropathic pains. Previous clinical studies have shown a positive effect of TENS analgesia in patients with osteoarthritis pain, low back pain and postoperative pain [11-13]. In inflammatory models of rats, TENS was also shown to significantly reduce pain sensitivity of both pressure and heat [14,15]. Furthermore, both high- and low-frequency TENS have been shown to cause hypoalgesia through the release of endogenous opioids in the CNS [16,17]. However, understanding of the mechanism of TENS analgesia from other way is still rare.

Recent findings from our group reveal that electroacupuncture (EA) treatment relieved inflammatory pain by inhibiting CFA-mediated activation of ERK1/2 in the SCDH [18]. It is generally thought that EA and TENS share the similar therapeutic effect on alleviating pain hypersensitivity. Therefore, in the current study, using the adjuvant-induced inflammation model,we sought to detemine the contribution of TENS to regulate the activation of ERK1/2 pathway in the SCDH, thus preventing early inflammatory pain.

## Methods

### Animals and CFA injection

Animal care, surgery, and handling procedures were approved by Zhejiang Chinese Medical University, and carried out in accordance with National Institutions of Health Guide for the Care and Use of Laboratory Animals in order to relieve suffering. Adult Male Sprague–Dawley rats (220 to 250 g) were obtained from the Department of Animal Sciences, Zhejiang Chinese Medical University. A total of 90 rats were housed under controlled temperature (23°C ± 1°C), relative humidity (70% ± 10%), and artificial 12-hour light–dark cycle lighting, with distill water and food available ad libitum. Rats were randomly divided into three groups: (a) the control group with saline injection (n = 14), with the same manner and volume as CFA injection; (b) the model group with CFA injection (n = 38); (c) the TENS group with CFA injection and TENS treatment (n = 38).

After baseline behavioral measurement, inflammatory pain rat model was induced by injection of 100 μl Complete Freud’s Adjuvant (CFA, sigma, USA) into the plantar surface of right hind paw. Rats were then returned to their cage and allowed to recover. 10 rats from each group were randomly chosen for behavioral testing. Furthermore, all rats were anesthetized with 10% choral hydrate (0.35 ml/100 g, i.p.) and perfused transcardially with 150 ml cold sterilized saline. Ipsilateral spinal dorsal horns (L_4_-L_6_) were removed, preserved at −80°C, and used for western immunoblotting (for p-ERK1/2 and COX-2), qPCR (for COX-2), and Enzyme-Linked Immunosorbent Assay (ELISA) (for PGE_2_).

### Behavioral testing

Paw volume were measured with a water plethysmometer (Plethysmometer 7140, UGO-Basile, Italy) at 3 d before the experiment (base) and 5 h, 6 h, 25 h after CFA administration. The paw withdrawal thresholds (PWTs) were performed as perivously destribed [19]. In brief, PWTs were tested with an automated von Frey-type testing device (Dynamic Plantar Aesthesiometer 37450, UGO Basile, Italy). Rats received two training sessions before the start of the experiment. Rats were placed on a metal mesh table and adapted to the new environment (30 min). The mechanical stimulus was delivered to the plantar surface of right hind paw below the floor of the plastic test chamber. A steel rod (0.5 mm diameter) was pushed against the hind paw with ascending force (from 0–50 g over a 20 sec period). When the animal withdrew its hind paw, the mechanical stimulus was automatically stopped and the force at which the rat withdrew its paw was recorded to the nearest 0.1 g. The sensitive of mechanical touch to the paws was measured at 3 d before the experiment (base), then 5 h, 6 h and 25 h after CFA administration.

### TENS treatment

Rats were loosely immobilized by assistants’ hands. TENS treatment (using two square self-adhesive electrodes [5 × 5 mm]) was administered to the physical denuded skin surround knee and leg of both right and left hind paws. The first electrode was applied between the tibia and fibula, 5 mm below the knee, and the other electrode was placed at the location 3 mm proximal to the lateral malleolus. When simulated, these sites have been shown to produce analgesia and reduced allodynia in a rat model of inflammatory pain [19], and were thus chosen for our study. Stimulation was delivered by the output terminals of the HANS Acupuncture Point Nerve Stimulator (LH-202H, Huawei co., Ltd., Beijing, China). TENS was delivered with the same parameters constant, constant square wave current output (pulse width: 0.6 ms at 2 Hz, 0.2 ms at 100 Hz); intensities ranging from 1–2 mA (each intensity for 15 min, totaling 30 min); at a 2 Hz and 100 Hz alternating frequencies (automatically shifting between 2 Hz and 100 Hz stimulation for three seconds each). The TENS stimulation was given at 5 h and 24 h after CFA injection. For eliminating the stress effect, rats in model group were also loosely immobilized by assistants’ hands as same as TENS group.

### Western blot analysis

Tissue sample were homogenized in lysis buffer, containing a cocktail of phosphatase inhibitors and proteinase inhibitors. The extracted protein was boiled in sodium dodecyl sulfate (SDS) sample buffer (100 mm Tris, ph 6.8, 2% SDS, 20% glycerol, 10%β-mecraptoehanol, and 0.1% bromophenol blue). Protein sample (20 μg per lane) separated by SDS-polyacrylamide gel electrophoresis and then transferred onto nitrocellulose membranes (Milliproe, USA). After membrane were blocked(1 h, 37°C)(tirs-buffered saline [TBS] with 0.2% Tween [TBST] and 5% skim milk), they were then incubated (overnight, 4°C) with the following primary antibodies: anti-p-ERK1/2 (1:2000, Cell Signaling Technology, USA), or anti-COX-2 (1:500, Caymen Chemical company, USA) in TBST. Membranes were then incubated (1 h at 37°C) with horseradish peroxidase-conjugated secondary antibody (1:10000) and, protein bands were visualized via ECL (Thermo scientific, USA) (1 min incubation) and exposed using the ImageQuant Las 4000 (General Electric company, USA). Band densities were quantified with Image Quant software (General Electric company, USA). β-actin (1:1000) (Cell Signaling Technology) was used as internal control.

### qPCR

Total RNA was extracted using Trizol Reagent (Invitrogen, France) containing guanidium thiocyanate, according to the manufacturer’s instructions. RNA was quantified by spectrophotometry. First strand cDNA was synthesized from 1 μg of total RNA (final volume of 10 μl) using the PrimerScript® RT reagent Kit with gDNA Eraser (TakaRa, Japan). Relative mRNA levels were quantified with RT-PCR using the fluorescent EvaGreen technology. cDNA was subjected to qPCR using the CFX96™ real-time PCR detection system (Bio-Rad, USA). Primer premier 5.0 software (Premier, Canada) was employed to design oligonucleotide primers specific for rat COX-2 and GAPDH (an internal control). COX-2: forward: 5′-CACGGACTTGCTCACTTTGT T-3’, reverse: 5′-AAGCGTTTGCGGTACTCATT-3′; GAPDH: forward: 5′-TGCTGAGTATGTCGTGGAG-3′; reverse: 5′-GTCTTCTGA GTGGCAGTGAT-3′, with the product sizes 161 bp, and 288 bp, respectively. Reactions (total volume, 20 μl) were incubated at 95°C for 3 min, followed by 40 cycles of 10 s at 95°C and 30 s at 55.9°C. Water controls were included to ensure specificity. Each sample was measured in triplicate, and data points were examined for integrity by analysis of the amplification plot. Adding the melting curve analysis in the reaction condition, the analytical model: 65°C-95°C, an increase of 0.5°C every 10 s. The comparative cycle threshold Cq method was used for relative quantification of gene expression. The amount of COX-2 mRNA normalized to the GAPDH and relative to a calibrator, was given by 2^-ΔΔCq^, with Cq indicating the cycle number at which the fluorescence signal of the PCR product crosses an arbitrary threshold set within the exponential phase of the PCR, and ΔΔCq = [(Cq_target (unknown sample)_-Cq_end.control (unknown sample)_)]-[(Cq_target (calibrator sample)_-Cq_end. control (calibrator sample)_)].

### ELISA

PGE_2_ was measured from extracted protein samples using a Parameter™ PGE_2_ Immunoassay ELISA kit (R&D Systems, USA), according to the manufacturer’s instructions. Each sample was examined in duplicate and averaged for data analysis.

### Statistical analysis

All data were expressed as means ± standard error mean (SEM). A repeated measures ANOVA with between-subjects factors was used to analyze paw volume and PWT data enabled, and a one-way ANOVA for independent samples to compare differences between groups at each time period. The post hoc test for least significant difference (LSD) was performed to determine differences between groups. Significance was reached at values of *P* < 0.05.

## Results

### Effect of TENS on paw volume in CFA rats

**Figure 1:**
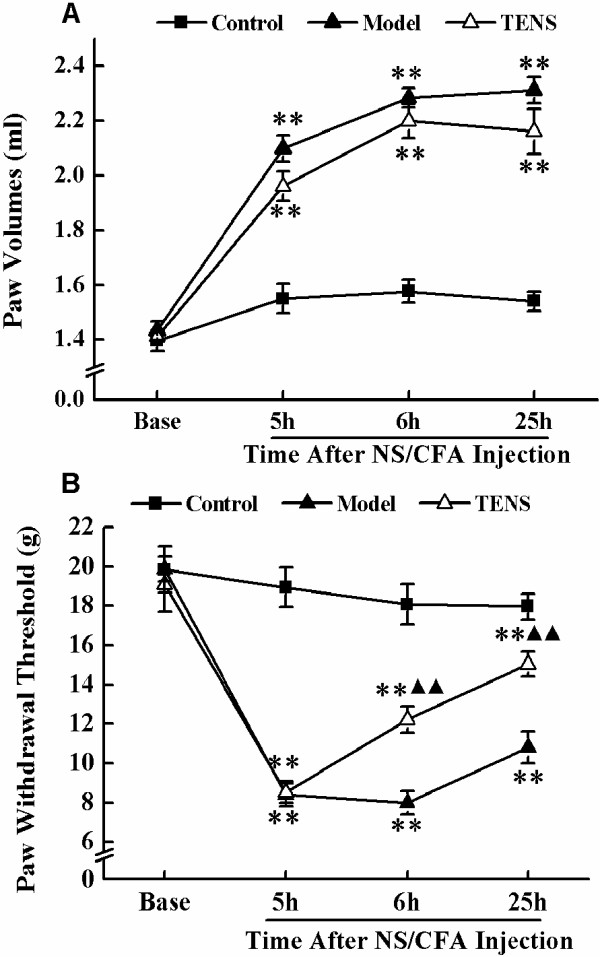
**Change of paw volume and paw withdrawal thresholds at base, 5 h, 6 h, and 25 h after NS/CFA injection in control group, model group, and TENS group. A**) Paw volumes were measured by a water plethysmometer. **B**) PWTs were assessed by using dynamic plantar aesthesiometer. Values represent mean ± SEM; n = 10 for per group. **P < 0.01 versus control group at the corresponding time point. ^▲▲^*P <* 0.01 versus model group at the corresponding time point.

All data of rats’ paw volume in every experimental group at each time point were shown in Figure 1A. The repeated-measures ANOVA with between-subjects factors revealed differences in paw volume over time points (*P* < 0.01) and between groups (*P* < 0.01). There was significant interactive effect between time points and groups (*P* < 0.01). Post-hoc LSD tests indicated there was no remarkable difference in the severity of paw volume of the whole process between the TENS group and the model group (*P* > 0.05).

### Effect of TENS on inflammatory pain behaviors in CFA rats

Mean PWTs in all experimental groups at each time point were shown in Figure 1B. The repeated-measures ANOVA with between-subjects factors revealed differences over time points (*P* < 0.01) and between groups (*P* < 0.01). There was significant interactive effect between time points and groups (*P* < 0.01). Post-hoc LSD tests indicated a significant analgesic effect in the TENS group when compared with the model group (*P* < 0.01). However, PWTs in TENS group still showed a significant difference compared with those in control group (*P* < 0.01).

At 5 h after CFA injection, the rats began to act overt behavioral sign, for instant, limping and guarding of the limb. One-way ANOVA for independent samples identified significant differences occurred among control, model and TENS groups from 5 h to 25 h after CFA injection. At the time points of 5 h, 6 h, and 25 h after CFA injection, the PWTs of the right hind paw in model and TENS groups were decreased significantly in comparison with that in control group (*P* < 0.01). Following the administration of treatment, PWTs in TENS group were significant higher than that in model group at 6 h and 25 h (*P* < 0.01). However, TENS group still kept a significant lower PWTs than control group, even at the ending of our study (*P* < 0.01).

### TENS administration inhibits p-ERK1/2 expression in the lumbar SCDH

**Figure 2:**
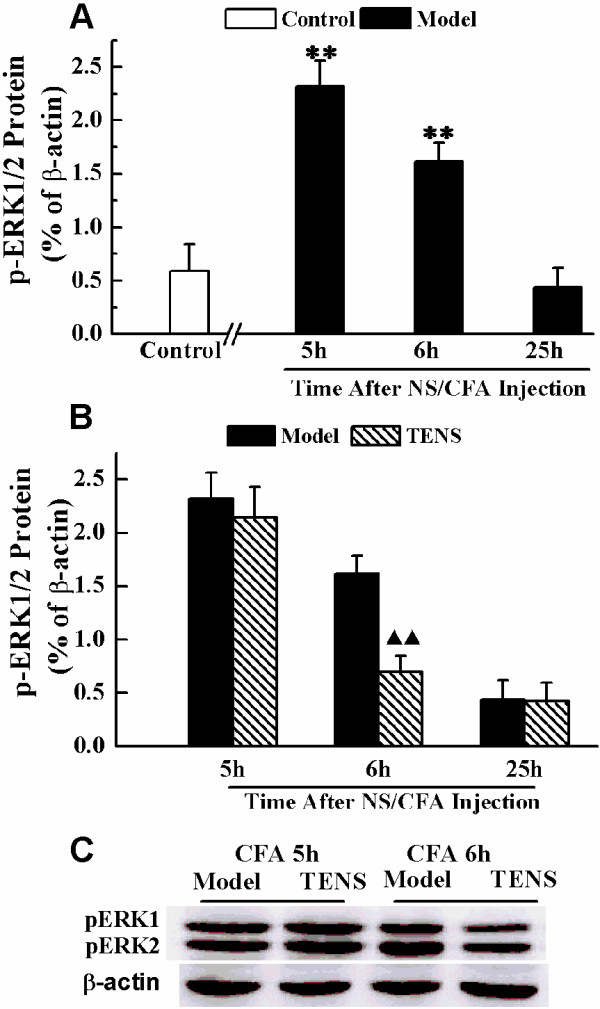
**Expression of p-ERK1/2 in the L**_**4**_**-L**_**6 **_**spinal cord dorsal horn at control, model and TENS groups after NS/CFA injection. A**) Statistical summary of the densitometric analysis of p-ERK1/2 expressed relative to the value of rats in control group. **B**) Statistical summary of the densitometric analysis of p-ERK1/2 expressed in rats of model group and TENS group at 5 h, 6 h and 25 h after CFA injection. **C**) Representative Western bolts show the level of p-ERK1/2 in rats model group and TENS group at 5 h and 6 h after CFA injection. Values represent mean ± SEM; n = 4-6 for model and TENS group at each time point; n = 4-6 for control group. **P < 0.01 versus control group at each time point. ^▲▲^*P <* 0.01 versus model group at the corresponding time point.

CFA injection produced localized swelling and mechanical allodynia, which persisted for the duration of the experiment (25 h). The peripheral inflammation induced by the CFA injection resulted in the induction of p-ERK1/2 in the superficial dorsal horn on the ipsilateral side of the L_4_-L_6_ spinal cord. The expression of p-ERK1/2 significantly increased at 5 h and remained higher at 6 h (*P* < 0.01), but significantly reduced at 25 h after CFA injection (Figure 2A). The reduction at 25 h promped us to test if TENS treatment regulated p-ERK1/2 from 5 h to 25 h after CFA injection. The level of p-ERK1/2 in TENS group at 6 h was decreased (*P* < 0.01) compared with those of rats in model group (Figure 2B). However, this effect was not observed at 25 h (*P* > 0.05) (Figure 2B).

### TENS administration inhibits expression of COX-2 in the lumbar SCDH

**Figure 3:**
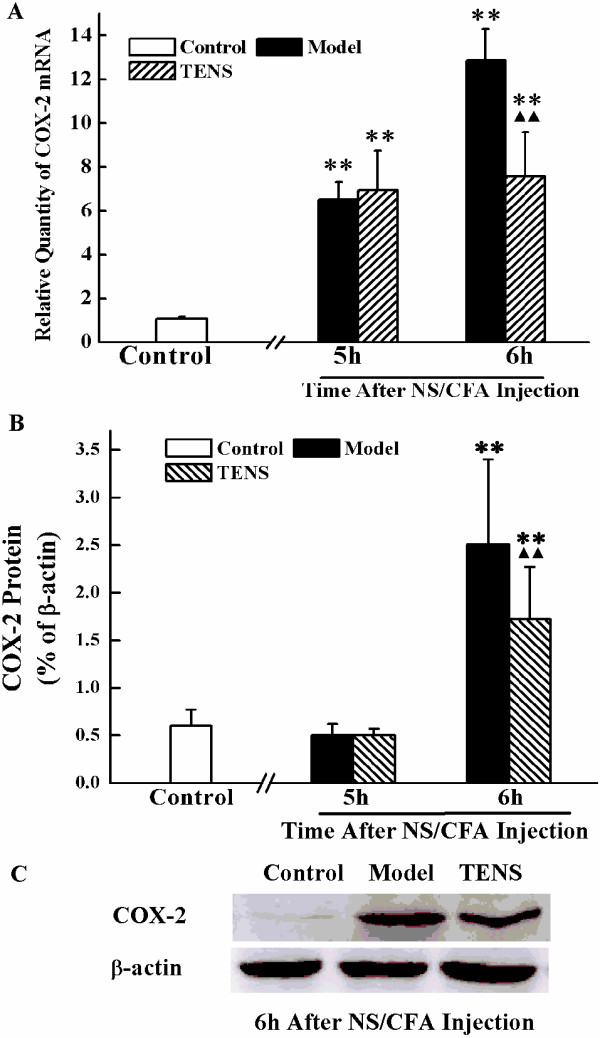
**Expression of COX-2 mRNA and protein in the L**_**4**_**-L**_**6 **_**spinal cord dorsal horn at control, model and TENS group after NS/CFA injection. A**) Statistical summary of the COX-2 mRNA expressed in spinal in rats of control group, model group and TENS group. **B**) Statistical summary of the densitometric analysis of COX-2 protein expressed in spinal in rats of control group, model group and TENS group. **C**) Representative Western bolts showed the level of COX-2 in rats of control, model and TENS group at 6 h after CFA injection. Values represent mean ± SEM; n = 4-6 for model and TENS group at each time point; n = 4-6 for control group. **P < 0.01 versus control group at the corresponding time point. ^▲▲^*P <* 0.01 versus model group at the corresponding time point.

We tested mRNA and protein expressions of COX-2 in SCDH, to investigate whether TENS regulates COX-2 expression for an analgesic effect. In CFA-treated rats, mRNA expression of COX-2 was significantly increased at 5 h and 6 h (*P* < 0.01) (Figure 3A). However, results differed at the post-translational level whereby expression of COX-2 protein was only increased at 6 h after CFA injection (*P* < 0.01) (Figure 3B). After once administration, TENS treatment to CFA-injected rats decreased both mRNA and protein expressions of COX-2 compared with CFA rats without TENS (*P* < 0.01) (Figure 3A, B). However, a significant difference in COX-2 mRNA and protein expressions were observed between control group and TENS group (*P* < 0.01) (Figure 3B).

### TENS regulating PGE_2_ level in the lumbar SCDH

**Figure 4:**
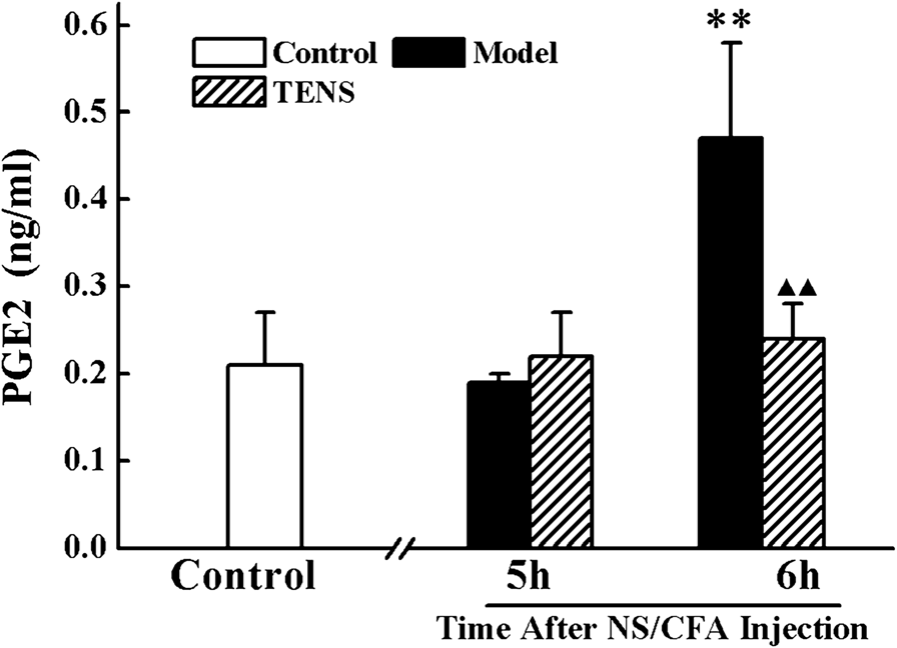
**Expression of PGE**_**2 **_**protein in the L**_**4**_**-L**_**6 **_**spinal cord dorsal horn at control, model and TENS group after NS/CFA injection.** Statistical summary of the PGE_2_ protein expressed in spinal dorsal horn in rats of control, model and TENS groups. Values represent mean ± SEM; n = 3-4 for model and TENS group at each time point; n = 3-4 for control group.**P < 0.01 versus control group at the corresponding time point. ^▲▲^*P <* 0.01 versus model group at the corresponding time point.

PGE_2_ levels were significantly increased in the CFA group compared with the control group at only 6 h post modeling (*P* < 0.01) (Figure 4). Exposure of CFA rats to TENS resulted in a significant reduction of the PGE_2_ levels when compared with those without TENS (*P* < 0.01). No significant difference was found between TENS group and control group at 6 h (*P* > 0.05).

## Discussion

The present study demonstrates that application of TENS at the hind paw attenuates inflammation-induced pain, furthermore inhibits the activation of ERK1/2, and up-regulations of COX-2 and PGE_2_ in SCDH.

Both peripheral inflammatory and central neuropathic mechanisms are involved in inflammatory pain [20]. ERK1/2 activated in SCDH neurons was shown to play an important role in pain hypersensitivity [21]. Zhuang et al. (2005) demonstrated that sequential activation of ERK1/2 in SCDH microglia and astrocytes was important for the induction and maintenance of neuropathic pain in rats with spinal nerve ligation [22]. Mounting evidence exists for the association of activated ERK1/2 in SCDH neurons and inflammatory pain [4-9], especially in CFA rat, in which p-ERK1/2 was shown to peak in 10 min, and remained elevated with a slowly decline for 48 h [5]. Furthermore, intrathecal injection of MEK inhibitors has been shown to inhibit inflammatory mechanical allodynia following hind paw injection of CFA [5]. In present study, p-ERK1/2 in the ipsilateral lumbar SCHD increased markedly at 5 h and 6 h after CFA injection. However, unlike other studies, there is no significant difference in p-ERK1/2 between the control and model groups when treated for 25 h. This lack of effect may have been a result of the activation of ERK1/2 in a small subset of dorsal horn neurons [5], to which western blot analysis would have thus been less sensitive in the detection of p-ERK1/2. Taken together, these results suggest that p-ERK1/2 plays an important role in decreased PWTs caused by peripheral inflammation, and inhibition of ERK1/2 activation may be a novel treatment for inflammatory pain.

Recent studies have reported that expression of spinal COX-2 mediates mechanical inflammatory pain hypersensitivity [23], which is reduced via the intrathecal injection of a COX-2 inhibitor [24]. Furthermore, COX-2 expression has been correlated with ERK1/2 activation, whereby inhibition of ERK1/2 activation blocked the producton of COX-2 production [25,26]. Our findings indicated high expression of COX-2 mRNA in SCDH at 5 h and 6 h after CFA injection, a finding previously observed [27]. However, COX-2 protein production at only 6 h indicates the lag time for its post-translational regulation. Numerous studies have indicated that ERK1/2 was likely to produce pain hypersensitivity via the inducing of expression of pronociceptive substance, such as COX-2 [28]. Therefore, the results from our study suggest that ERK1/2-COX-2 pathway contributes to the inflammatory pain hypersensitivity in SCDH.

TENS is a non-pharmacologic and noninvasive treatment for pain, commonly used in patients with acute and chronic pain. TENS has been shown to be effective for osteoarthritis, rheumatoid arthritis, and postoperative pain [29-31] and can relieve mechanical allodynia in animal models of joint, muscle, and cutaneous inflammation [32-34]. TENS was applied with varying frequencies, from 2 Hz (low) to 100 Hz (high) and different frequencies led to different analgesic effects [35]. In the study, the effect of TENS with alternating frequencies (2Hz/100Hz) on inflammatory pain induced by CFA injection was evaluated. Our findings that TENS produces analgesic effect at 6 h after CFA injection are consistent with previous findings that TENS partially reversed the hyperalgesia at 4 h after carrageenan-induced paw inflammation [36]. Despite TENS-meditated analgesia, we did not detect an anti-inflammatory effect, suggesting that TENS may inhibit the inflammatory pain hypersensitivity independent of its anti-inflammatory action.

Our previous study has told that EA, at the ST36 acupoint, inhibited the expression of p-ERK1/2 and p-p38 MAPK in ipsilateral SCDH, and also induced a hyperalgisic response [18,37]. These results suggested that the modulation of MAPK activation in SCDH as an underlying mechanisms of EA-mediated inhibition of pain. Based on current literature, the basic mechanisms of TENS- and EA-mediated analgesia are similar, however, the effects of TENS on ERK1/2 activation remain unknown, particularly at the spinal level. In the present study, our findings verified that, in addition to the modulation of PWTs, TENS treatment significantly decreased the expression levels of p-ERK1/2 and COX-2 in SCDH at 6 h after CFA injection. Previous studies at the spinal level have shown that TENS-mediated reduction of pain hyperalgesia is regulated by the release of gama-aminobutyric acid (GABA) and decreaed glutamate levels [38,39], in addition to endogenous opioid signaling [17]. Furtermore, TENS mediated reduction of hyperalgsia by reducing the sensitization of dorsal horn neurons through regulating GABA and glutamate receptors [40]. Glutamate transmission via NMDA receptors was shown to be essential for ERK1/2 activation in SCDH neurons [41] and its contribution to central sensitization [42]. Moreover, neuronal expression of COX-2 in the spinal cord facilitated the development of a central component of inflammatory pain hypersensitivity via increasing neuronal excitation and reducing inhibition [23,43]. Overall, regulation of the ERK1/2-COX-2 pathway in SCDH may be the signaling transudation pathway underlying the TENS-mediated analgesia.

In order to verify the speculation that inhibition of the activation of ERK1/2-COX-2 pathway may be the signaling transudation pathway underlying the TENS-mediated analgesia, protein level of PGE_2_ in SCDH were detected by ELISA. Increased PGE_2_ in the CNS after peripheral inflammation mediated a widespread increase in mechanical pain sensitivity due to synaptic facilitation within the spinal cord [43]. Furthermore, the source of PGE_2_ is predominantly via COX-2 activation [44]. Our findings reveal that like the COX-2, the protein level of PGE_2_ only increased at 6 h after CFA injection, and TENS significantly decreased the over-producation of PGE_2_ in SCDH. The ERK1/2-COX-2 pathway contributes to inflammatory mechanical allodynia [28], and COX-2 itself causes pain sensitivity by increasing PGE_2_ level in SCDH. Therefore, TENS may alleviates pain hypersensitivity by inhibiting ERK1/2-COX-2 pathway activation.

Other MAPK families associated with inflammatory pain may also play a role [1], and thus the effect of TENS on other signal transduction may provide additional novel therapeutic targets. To further elucidate the mechanisms of TENS-mediated analgesia, future studies could focus on other MAPK families and inflammation-induced thermal hyperalgesia.

## Conclusions

TENS-mediated analgesia to control peripheral inflammatory pain is independent of anti-inflammatory activity. Furthermore, CFA-induced activation of the ERK1/2-COX-2 pathway in SCDH neurons plays an important role in developing and maintaining inflammatory mechanical allodynia. Taken together, the analgesic effect of TENS on inflammatory pain may be associated with the inhibition of the activaiont of the spinal ERK1/2-COX-2 pathway.

## Competing interests

The authors declare that they have no competing interests.

## Authors' contributions

JQF designed and performed experimental protocols described in this manuscript as well as the writing of the initial draft of the manuscript. FJF performed the Western blotting, tissue fractionation and associated analyses. YL provided supervision for data analysis, study direction, image acquisition, manuscript design and revisions. JYD performed experiments, contributed to the design, data analysis and writing of the manuscript. All of the authors have read and approved the final manuscript.

## Pre-publication history

The pre-publication history for this paper can be accessed here:

http://www.biomedcentral.com/1472-6882/13/134/prepub
